# Periodontitis and Rheumatoid Arthritis: The Same Inflammatory Mediators?

**DOI:** 10.1155/2019/6034546

**Published:** 2019-05-05

**Authors:** Fulvia Ceccarelli, Matteo Saccucci, Gabriele Di Carlo, Ramona Lucchetti, Andrea Pilloni, Nicola Pranno, Valeria Luzzi, Guido Valesini, Antonella Polimeni

**Affiliations:** ^1^Department of Internal Medicine and Medical Specialties, Sapienza University of Rome, Rome, Italy; ^2^Department of Oral and Maxillo-Facial Sciences, Sapienza University of Rome, Viale Regina Elena 287a, 00161 Rome, Italy

## Abstract

The strict link between periodontitis (PD) and rheumatoid arthritis (RA) has been widely demonstrated by several studies. PD is significantly more frequent in RA patients in comparison with healthy subjects: this prevalence is higher in individuals at the earliest stages of disease and in seropositive patients. This is probably related to the role of *P. gingivalis* in inducing citrullination and leading to the development of the new antigens. Despite the many studies conducted on this topic, there is very little data available concerning the possibility to use the same biomarkers to evaluate both RA and PD patients. The aim of the review is to summarize this issue. Starting from genetic factors, data from literature demonstrated the association between HLA-DRB1 alleles and PD susceptibility, similar to RA patients; moreover, SE-positive patients showed simultaneously structural damage to the wrist and periodontal sites. Contrasting results are available concerning other genetic polymorphisms. Moreover, the possible role of proinflammatory cytokines, such as TNF and IL6 and autoantibodies, specifically anticyclic citrullinated peptide antibodies, has been examined, suggesting the need to perform further studies to better define this issue.

## 1. Introduction

Abundant evidences recorded in literature demonstrated that rheumatoid arthritis (RA) and periodontitis (PD) are frequently associated with each other and share several pathogenic and clinical features [1].

RA is a systemic inflammatory autoimmune disease characterized by chronic inflammation and joint tissue destruction, leading to functional disability. Similarly, PD patients experience chronic inflammatory diseases [1–3]. In particular, PD are dysbiotic conditions characterized by an imbalance between subgingival communities and host immune response. The transition from health to PD is characterized by shifts in the community structure of the complete subgingival microbiome [4–6].

PD is currently considered a risk factor for RA: the first link between these two conditions was identified in *P. gingivalis*, a gram-negative anaerobe bacteria characterized by the presence of peptidylarginine deiminase (PAD). This enzyme contributes to RA development by catalyzing citrullination, a posttranslational modification playing a crucial role in the production of anticyclic citrullinated peptide antibodies (ACPA), widely recognized as diagnostic and prognostic biomarkers for RA patients [7, 8]. Recently, our group observed a significant association between the percentages of *P. gingivalis*, assessed by real-time PCR, on the total tongue biofilm and RA disease activity (evaluated as disease activity score on 28 joints—DAS28) [9]. This result suggests that the oral cavity microbiological status could play a role in the pathogenic mechanisms of inflammation, leading to more active diseases [9, 10].

More recently, the role of *Aggregatibacter actinomycetemcomitans* has been suggested. This oral pathogen could induce hypercitrullination at neutrophil level by secreting leukotoxin A that is able to change neutrophil morphology, mimicking extracellular trap formation. Finally, this process results in the hypercitrullinated autoantigen release, triggering autoimmune response in RA patients [11]. From an epidemiological point of view, several studies have been conducted which remarked the association between RA and PD. The main data is summarized in Table 1. Specifically, case-control studies underline a higher prevalence of PD in RA patients in comparison with healthy controls [12–24]. PD prevalence is higher in early RA patients at disease onset, despite the young age and paucity of smoking history [14–18]. In addition to higher prevalence, RA patients show a more aggressive PD compared to HS (healthy subjects) [15, 16, 18–21]. Furthermore, some studies have carried out a comparison with OA patients. Dissick and his colleagues observed that PD was more common and severe in RA patients compared to OA. Moreover, in RA, the presence of PD was significantly associated with positivity for rheumatoid factor (RF) and ACPA [13]. More recently, Gonzalez and coauthors observed that ACPA-positive RA patients showed significantly higher mean percentage of sites with alveolar bone loss (ABL) greater than 20% in comparison with OA patients. Moreover, ABL substantially connected to ACPA titers and disease activity in terms of DAS28 [25]. The same cohort was evaluated in terms of HLA-DRB1 and anti-*P. gingivalis* antibodies, showing higher ACPA levels in patients with subgingival *P. gingivalis* and in those with higher anti-P. gingivalis antibody levels [26].

Moving passed the strict association between RA and PD, from an epidemiological and pathogenic point of view, it is possible to hypothesize a common genetic background and the sharing of inflammatory mediators between RA and PD. The review thoroughly covers this topic.

### 1.1. Genetic Biomarkers

Several studies have confirmed the role of genetic factors in the RA development: according to a multifactorial model, the interaction between genetic background and environmental factors leads to the development of an autoimmune inflammatory condition, resulting in autoantibodies production. The highly polymorphic HLA-DRB1 locus (the so-called shared epitope—SE) represents the strongest genetic factor involved in disease development. Particularly, all HLA-DRB alleles with the SE provide RA-prone antigen recognition: this leads not only to an increased risk of developing RA but also to the progression into a more erosive, deforming disease. Furthermore, a gene–environment interaction between smoking and SE genes seems to be crucial in the development of seropositive RA. Nonetheless, the contribution of other genetic polymorphisms on RA susceptibility has been also investigated: among these, SNPs in signal transducer and activator of transcription 4 (STAT4), Fc gamma receptor (FCGR), protein tyrosine phosphatase nonreceptor type 22 (PTPN22), PADI-4, tumor necrosis factor (TNF), and interleukin 6 (IL6) genes have been associated with disease development in several case-control studies [27]. It is important to note that genetic polymorphisms could be also associated with different disease phenotypes, in terms of radiographic damage progression [28–30]. Moreover, in 2011, we suggested the possible role of TGF-*β* 869C/T and IL6-174G/C polymorphisms in determining erosive damage evaluated by ultrasonographic assessment in a cohort of RA patients [31].

Some of these genetic factors have been also associated with PD susceptibility, reinforcing the hypothesis of common pathogenic mechanism with RA. Specifically, SE positivity has been widely linked to PD development.

In 2006, Marotte and colleagues investigated the presence of an association between bone destruction at the joint and periodontal level in a wide RA cohort. The analysis of 147 subjects—56.5% of whom with PD—demonstrated a strong association between PD and wrist destruction, assessed by the radiographic Larsen score. Specifically, the authors identified a significant association between SE positivity and bone destruction in wrist and periodontal sites. In fact, SE+ patients showed 2.5 times greater risk of having wrist joint destruction than SE- (OR = 2.5). In the same way, SE+ patients had a 2.2 times greater risk to have periodontal destruction compared to SE- (OR = 2.2). The comparison between patients with both site destruction and those without any destruction demonstrated the association with SE positivity (OR = 3.9). This evidence underlines the possible role of SE in bone destruction at both sites, suggesting a simultaneous action [32].

Data from Marotte and colleagues agreed with a previous study conducted by Bonfil and colleagues in 1999, suggesting the role of SE as a prognostic factor for PD susceptibility [33].

The possible role of SE-coding DRB1 alleles has been recently underlined by Gehlot and colleagues: the authors observed that transgenic SE+ mice, but not SE- mice, spontaneously developed PD, associated with IL17 overexpression and periostin disruption. Moreover, SE-positive mice showed significantly lower mandibular bone volumetric and mineralization parameters, together with increased alveolar bone resorption [34].

In addition to SE, the possible role of other RA-related genetic polymorphism has been investigated to analyze the association with bone destruction at periodontal level [35]. The studies conducted so far did not produce conclusive results, mainly due to small size cohorts (generally less than 100 patients enrolled), leading to a lack of statistical power to properly detect an association. Hereupon, genotype and allele frequencies could widely vary between different ethnic groups and the same genetic variants could play a different role in different populations. Data from literature provides some evidences to support the association between an aggressive PD and SNPs in interleukin 1 beta (IL1*β*), interleukin 1 receptor antagonist (IL1RN), FCGR IIIb, vitamin D receptor (VDR), and Toll-like receptor 4 (TLR4) genes. Moreover, a chronic PD was associated with polymorphisms in IL1B, IL1RN, IL6, IL10, VDR, CD14, TLR4, and matrix metalloproteinase-1 (MMP1) genes [34]. The low statistical power of these studies was also demonstrated by the results of the meta-analysis conducted by Nikolopoulos and colleagues in 2008, confirming exclusively a moderate and weak positive association between the IL1 composite and IL1B-511 genotypes and the occurrence of chronic PD [36]. More recently, the SNP rs2237892 of KCNQ1 gene resulted in a significant association with the coexistence of RA and chronic PD, confirmed in the multiple logistic regression. These results suggest that individuals carrying rs2237892 T allele are likely to have both diseases [37]. The specific role of KCNQ1 gene in RA and PD pathogenesis has not been completely defined: the associated SNP is located in intron 15 of the KCNQ1 gene on chromosome 11p 15.5, encoding the pore-forming *α* subunit of a voltage-gated K^+^ channel, crucial for the repolarization phase in the cardiac muscle. Moreover, this channel is also expressed at the plasma membrane of fibroblast-like synoviocytes from RA patients and could play a role on cell proliferation and adhesion and secretion of proinflammatory cytokines [38].

### 1.2. Inflammatory Biomarkers

As widely demonstrated, both RA and PD are characterized by an imbalance between proinflammatory and anti-inflammatory cytokines. In general, high levels of IL1, IL6, and TNF have been demonstrated both in patients with RA and PD. This increased expression of proinflammatory cytokines could stimulate STAT3 activation, playing a key role in the pathophysiology of RA and PD [39].

Particularly, an increased expression of IL1 and TNF has been demonstrated in RA synovium and PD gingival tissues [40]. The central role of inflammatory cytokines in RA pathogenesis has been confirmed by the introduction of biological drugs more than 20 years ago. These drugs are characterized by an innovative mechanism of action, based on the targeted inhibition of specific molecular or cellular targets directly involved in the disease pathogenesis: proinflammatory cytokines (TNF, IL1, and IL6), CTLA-4, and molecules involved in the activation, differentiation, and maturation of B cells. Their use is associated with better prognosis and the possibility to obtain a clinical remission [41].

Moving on a PD scenario, increased IL1 and TNF levels in periapical exudates were identified in these patients [42]. Moreover, PD progression was reduced by IL1 and TNF inhibitors in experimental models: specifically, histomorphometric analysis indicates that IL1 and TNF antagonists significantly reduced the loss of connective tissue attachment by approximately 51% and the loss of alveolar bone height by almost 91% [43]. In 2013, Cetinkaya and colleagues aimed to assess whether PD and RA patients share similar proinflammatory and anti-inflammatory cytokine profiles at a serum and gingival crevicular fluid (GCF) level. The study included 17 RA patients, 16 PD patients, and 16 HS. The authors did not obtain consistent results regarding proinflammatory and anti-inflammatory cytokine levels. Specifically, the total amount and GCF concentration of IL1b, IL4, IL10, and TNF were similar in RA and PD patients. However, the authors underlined the possible influence of the treatment in RA patients [44].

The salivary levels of matrix metalloproteinase-8 (MMP8) and IL1B were also evaluated in RA patients in comparison with PD and HS. The PD group showed significantly higher salivary levels of MMP-8 and IL1B in comparison with other groups; nevertheless, IL1B was the only biomarker significantly higher in RA compared to controls. Interestingly, RA patients treated by anti-TNF showed lower IL1B and TNF levels compared to nontreated patients [45].

Despite these nonconclusive results, some studies suggest that anticytokine treatment could improve PD. In 2016, Kobayashi and colleagues demonstrated a significant reduction of periodontal inflammation (assessed in terms of gingival index, bleeding on probing, and probing depth) in RA patients treated with tocilizumab and TNF inhibitors. As expected, treatment induced also decreased significantly in RA disease activity parameters, including DAS28-CRP, number of tender and swollen joints, and serum levels of ACPA and RF [46]. This treatment relationship was confirmed also by the evidence that nonsurgical treatment for PD seems to be able to improve the RA activity status: growing evidences demonstrated significant improvement of ESR, CRP, and DAS28 during PD treatment in RA patients [47, 48].

## 2. Autoantibodies

Some studies have investigated the presence of RA-related autoantibodies in PD patients. Taken together, PD patients demonstrated a high frequency of ACPA compared to controls; moreover, these antibodies showed a significantly higher titer in PD [3, 49].

However, these studies enrolled small populations and differences between patients and controls were not statistically significant. In 2014, De Pablo and colleagues tested sera from 194 patients with and without PD, none of whom with RA, to assess the presence of different antibodies. PD was associated with a normal frequency of ACPA and antimutated citrullinated vimentin (about 1%), but a significantly higher frequency of positive anticitrullinated *α*-enolase peptide-1 (anti-CEP-1; 12% versus 3%) and its uncitrullinated form (16% versus 2%; *p* < 0.001). Moreover, positive antibodies against uncitrullinated fibrinogen and uncitrullinated equivalent of CCP were more common in PD compared to non-PD patients (26% versus 3%; 9% versus 3%). The presence of these autoantibodies was not associated with smoking status, confirming that the PD autoantibody response was not exclusively due to smoking [50]. Moreover, the study conducted by Gonzales in 2015 underlines the possible role of ACPA, observing that ACPA-positive RA patients had a significantly higher mean percentage of sites with ABL greater than 20% compared to OA controls [51]. More recently, the presence of anticitrullinated histone H3 autoantibodies was investigated: these biomarkers were found in 39% of RA patients compared to 8% in HC and 10% in PD patients. No associations were found between anticitrullinated histone H3 levels and periodontal status in RA patients [52].

## 3. Conclusions

**Figure 1:**
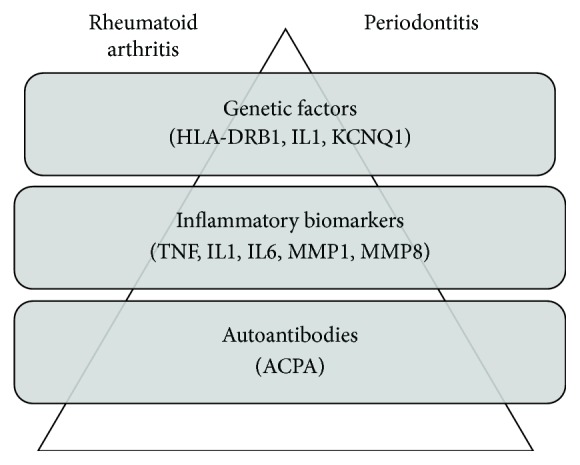
Suggested genetic factors, inflammatory biomarkers, and autoantibodies shared by rheumatoid arthritis and periodontitis. IL1: interleukin-1; TNF: tumor necrosis factor; MMP1: matrix metalloproteinase-1; MMP8: matrix metalloproteinase-8; ACPA: anticyclic citrullinated peptide antibodies.

Despite the widely demonstrated connection between RA and PD from an epidemiological and pathogenetic point of view, data from literature does not seem to support the sharing of the same mediators. Regarding genetic factors, the most consistent data are related to HLA-DRB1 alleles: especially the presence of SE is associated with susceptibility and severity in both diseases. Conversely, contrasting results are available concerning other genetic polymorphisms or proinflammatory cytokines such as TNF, IL1, and IL6. Finally, there are few cases in which RA-related antibodies are found in PD patients. These evidences are graphically represented in Figure 1. Altogether, the paucity of data on this topic suggests that deeper studies, including wider populations and with a longitudinal design, are required to better clarify this issue.

## Figures and Tables

**Table 1:** Main study evaluating prevalence of periodontitis in rheumatoid arthritis patients.

Study	RA group (*N*)	Control group	Mean age (years)	Female sex (%)	Smokers in RA (%)	Smokers in controls (%)	RA duration	RF (%)	ACPA (%)	PD prevalence in RA	Results
Pischon et al. 2008 [12]	57	HS 52	RA 52.1	RA 95%	59.7%	40.4%	10 years	NR	NR	8.05-fold increased odds of PD in RA compared with HS	Higher prevalence of PD in RA versus HS
Dissick et al. 2010 [13]	91	OA 41	RA 62 OA 58	RA 12 OA 5	65%	46%	14 years	81%	87%	RA 51% OA 26%	PD more common and severe in RA pt in comparison with OA. Association between PD and RF/ACPA
Scher et al. 2012 [14]	eRA 31 lRA 34	18	eRA 42.2 lRA 47.7	eRA 68% lRA 79% HS 65%	eRA 32% lRA 30%	22%	eRA 3.4 mts lRA 62.9 mts	eRA 92% lRA 78%	eRA 96% lRA 88%	eRA 88% lRA 91% HS 44%	High PD prevalence in eRA at disease onset
de Smit et al. 2012 [15]	95	Non -RA 44 HS 36	RA 56 Non-RA 54 HS 34	RA 68 Non-RA 57 HS 57	24.2%	Non-RA 61.4% HS 38.9%	7.4 years	RA 53%	RA 71%	RA 43% moderate and 27% severe Non-RA 18% HS 12%	Higher prevalence of severe PD in RA pt in comparison with controls. Association between severe PD and higher DAS28
Ranade and Doiphode 2012 [16]	RA 40	Non-RA 40	RA 45	RA 80%	NR	NR	2.15 years	NR	NR	RA 97.5%	High prevalence of mild-to-moderate PD in patients with RA
Reichert et al. 2013 [17]	RA 42	Non-RD 114	RA 56.1	RA 52.4%	26.2%	25%	NR	NR	NR	RA 34.3% Non-RD 49.1%	In patients with RA, DNA of P. gingivalis was detected in synovial fluid more often than in controls
Wolff et al. 2014 [18]	eRA 22	HS 22	RA 51.7	RA 68%	19%	19%	5.9 mts	37%	41%	100%	More severe PD detected in eRA pts
Joseph et al. 2013 [19]	RA 100	HS 112	RA 46.5	RA 76%	0%	0%	NR	NR	NR	RA 58% HS 7.1%	Higher prevalence and severity of PD in RA
Chen et al. 2013 [20]	RA 13779	Non-RA 137790	RA 52.6	RA 77.4%	NR	NR	NR	NR	NR	RA 39% Non-RA 35.1%	Association between periodontitis and incident RA
Mikuls et al. 2014 [26]	287	OA 330	RA 59 OA 59	RA 37% OA 30%	62%	46%	12.6 years	77%	83.6%	RA 34.8% OA 26%	Higher PD prevalence in RA versus OA. PD significantly associated with higher disease activity, radiographic damage, and ACPA levels. Higher ACPA in pts with subgingival P gingivalis and in those with higher levels of anti-P gingivalis antibodies. No differences between RA and OA in the levels of anti-PG
Gonzalez et al. 2015 [25]	287	OA 330	RA 59 OA 59	RA 37% OA 40%	62%	46%	Not specified	Not specified	80.5%	100%	ACPA-positive RA patients with significantly higher mean percentage of sites with ABL >20% compared with OA pts
Potikuri et al. 2012 [22]	91	93	RA 43.9 HS 41.7	RA 76% HS 69%	0	0	PD 17.1 mts Non-PD 12.9 mts	63%	41%	RA 64.8% HS 28%	Strict association between PD and RA in nonsmoking subjects and DMARD-naïve pts
Eriksson et al. 2016 [21]	RA 2740	HS 3942	18-70 years	RA 73% HS 73%	25%	18%	9.6 years	64%	63%	RA 33% HS 32%	No evidence of an increased prevalence of PD in patients with lRA compared to HS and no differences based on ACPA or RF status among RA subjects
Choi et al. 2016 [23]	RA 264	HS 88	58.2	RA 87.5% HS 87.5%	6.4%	8%	13.8 years	68.5%	69.1%	RA 63.6 HS 34.1%	Prevalence of moderate or severe PD increased in RA patients compared to HS. Periodontal inflammation was correlated with RA duration, ESR, and ACPA
Äyräväinen et al. 2017 [24]	eRA 53 lRA 28	HS 43	eRA 51 lRA 52	eRA 85% lRA 82% HS 88%	eRA 21% lRA 11%	14%	eRA 10.4 mts lRA 176 mts	eRA 79.2% lRA 69.2%	NR	eRA 67.3% lRA 64.3% HS 39.5%	Moderate PD more frequent in RA patients than HS

RA: rheumatoid arthritis; PD: periodontics; HS: healthy subjects; OA: osteoarthritis; eRA: early RA; lRA: long-standing RA; ACPA: anticyclic citrullinated peptide antibodies; ABL: alveolar bone loss; ESR: erythrocyte sedimentation rate; mts: months; NR: not reported.
